# Increased Risk of Atrial and Ventricular Arrhythmia in Long-Lasting Psoriasis Patients

**DOI:** 10.1155/2013/901215

**Published:** 2013-04-03

**Authors:** Hakki Simsek, Musa Sahin, Aytac Akyol, Serkan Akdag, Hatice Uce Ozkol, Hasan Ali Gumrukcuoglu, Yilmaz Gunes

**Affiliations:** ^1^Cardiology Department, Faculty of Medicine, Yuzunci Yil University, 65200 Van, Turkey; ^2^Van High Edücation and Research Hospital, 65100 Van, Turkey; ^3^Dermatology Department, Faculty of Medicine, Yuzunci Yil University, 65200 Van, Turkey; ^4^Cardiology Department, Hisar Intercontinental Hospital, 34768 Istanbul, Turkey

## Abstract

*Background*. Several reports have demonstrated an association between psoriasis and cardiovascular diseases. P wave dispersion (PWD) is the most important electrocardiographic (ECG) markers used to evaluate the risk of atrial arrhythmias. QT dispersion (QTD) can be used to assess homogeneity of cardiac repolarization and may be a risk for ventricular arrhythmias. *Aim*. To search PWD and QTD in patients with psoriasis. *Methods*. Ninety-four outpatient psoriasis patients and 51 healthy people were evaluated by physical examination, 12-lead ECG, and transthoracic echocardiography. Severity of the psoriasis was evaluated by psoriasis area and severity index (PASI). *Results*. Mean disease duration was 129.4 ± 83.9 (range, 3–360) months and PASI ranged from 0 to 34.0 (mean ± SD; 7.6 ± 6.7). Compared to control group, psoriatic patients had significantly shorter Pmax and Pmin durations, longer QTcmax, and greater PWD and QTcD. Transmitral deceleration time (DT) and isovolumetric relaxation time (IVRT) were significantly longer among psoriasis patients. QTcD and PWD were significantly correlated with disease duration (*r* = 0.693, *P* < 0.001, and *r* = 0.368, *P* = 0.003, resp.). *Conclusions*. In this study, we found that both PWD and QTcD are increased in psoriasis patients compared to healthy subjects. In addition, they had longer DT and IVRT.

## 1. Introduction

Psoriasis is one of the most prevalent T cell-mediated chronic inflammatory disease affecting around 1–3% of human population worldwide [1]. Cardiovascular abnormalities like arterial hypertension, arterial atherosclerosis, and heart valve abnormalities are frequently observed in the course of this disease [2–5]. Although the pathogenesis of psoriasis is still not fully understood, psoriasis is associated with markers of systemic inflammation, such as increased C-reactive protein levels [6] and T-helper cell type 1 (TH1) cytokines [7], and inflammatory processes and oxidative stress are frequently delineated [8, 9]. Rhythm and conduction disturbances and sudden cardiac death are important manifestations of cardiac involvement in autoimmune rheumatic diseases with increased inflammation. However, there is scarcity of data on rhythm abnormalities and conduction disturbances in psoriatic patients.

P wave dispersion (PWD) is an electrocardiographic marker that has been associated with inhomogeneous and discontinuous propagation of sinus impulses [10, 11]. Prolonged P wave duration and increased PWD have been reported to carry an increased risk for AF [11, 12]. Recent studies have also demonstrated the possible implication of inflammation and oxidative stress in pathogenesis of AF [13–15].

QT dispersion (QTd) can be used to assess the homogeneity of cardiac repolarization and autonomic function [16]. Increased heterogeneity of repolarization has been associated with increased risk of ventricular tachyarrhythmias [17]. Kowalewski et al. [18] showed that levels of proinflammatory cytokines are increased in young people with ventricular arrhythmias having no evidence of myocardial injury suggesting an inflammatory background for ventricular arrhythmias.

Although there is no direct evidence for an association between psoriasis and AF or ventricular arrhythmias, we assume that systemic inflammation in patients with psoriasis might contribute to the development of arrhythmia. 

To the best of our knowledge, PWD and QTD in psoriasis have not been searched in the literature. In this study, we evaluated PWD and QTD in patients with psoriasis without overt cardiac involvement.

## 2. Methods

Ninety-four outpatient psoriasis patients and 51 healthy people were included in the study. Patients having systemic hypertension, diabetes mellitus, history of ischemic heart disease, significant valvular heart disease, chronic obstructive pulmonary disease, hypo- or hyperthyroidism, renal failure or any associated systemic disease were excluded. Diagnosis of psoriasis was confirmed by dermatological examination and/or punch biopsy. Severity of the psoriasis was evaluated by psoriasis area and severity index (PASI). Patients were evaluated by physical examination, 12-lead electrocardiography, and transthoracic echocardiography. The study was approved by the Yuzuncu Yil University, Faculty of Medicine Ethics Committee in accordance with Declaration of Helsinki. All participants were informed about the study and their consents were obtained. 

Twelve-lead ECGs were obtained after a 10-minute rest, with 20 mm/mV amplitude and 50 mm/s rate with standard lead positions between 13:00 and 16:00 o'clock using a commercially available machine (Marquatte Case, Hellige Medical System, Cardiosmart, Hellige Instrument Company, Freiburg, Germany). ECGs were manually measured by the use of a magnifying glass by two blinded cardiologists having no information about the patients. The beginning of the P wave was defined as the point where the initial deflection of the P wave crossed the isoelectric line, and the end of the P wave was defined as the point where the final deflection of the P wave crossed the isoelectric line. The difference between maximum and minimum P wave durations was defined as PWD. QT intervals were taken beginning from the onset of the QRS complex to the end of the T wave, which was defined as its return to the TP baseline. If U waves were present, the QT interval was measured to the nadir of the curve between the T and U waves. The R-R interval was measured and used to compute the heart rate and to correct QT interval (QTc) with Bazett's formula. QTc dispersion (QTcD) was determined as the difference between the maximum and minimum QTc intervals in different leads. No patient had less than nine measurable leads. The intraobserver and interobserver variations for PWD and QTcD were less than 5%.

All the study population underwent a transthoracic examination. Using M-mode echocardiography, long-axis measurements were obtained at the level distal to the mitral valve leaflets according to the recommendations of the American Society of Echocardiography. Pulse wave Doppler measurements of aortic and transmitral valve flow profiles were obtained. The transmitral flow velocity was measured using pulse wave Doppler with the sample volume positioned between the mitral leaflet tips during diastole. Early diastolic flow, atrial contraction signal, early diastolic flow/atrial contraction signal (E/A), and deceleration time (DT) were measured. Isovolumetric relaxation time (IVRT) was determined as the interval between the end of the aortic outflow and the start of the mitral inflow signal. Values were measured on three separate beats and then averaged for all parameters. Pulmonary artery systolic pressure (PASP) was calculated from tricuspid insufficiency flow in the parasternal short axis and apical four-chamber view, and the highest tricuspid regurgitation velocity was taken as a study sample. Estimated PASP above 30 mmHg was an exclusion criteria.

## 3. Statistics

Results are expressed as mean ± SD. Using an SPSS package 18.0, data between the groups were compared with Student's  *t*-test for continuous variables, chi-square test for qualitative variables, and Mann-Whitney  *U*-test for variables without normal distribution. Pearson correlation analysis was used to assess the correlation between variables. A two-tailed *P* value <0.05 was considered significant.

## 4. Results

**Figure 1:**
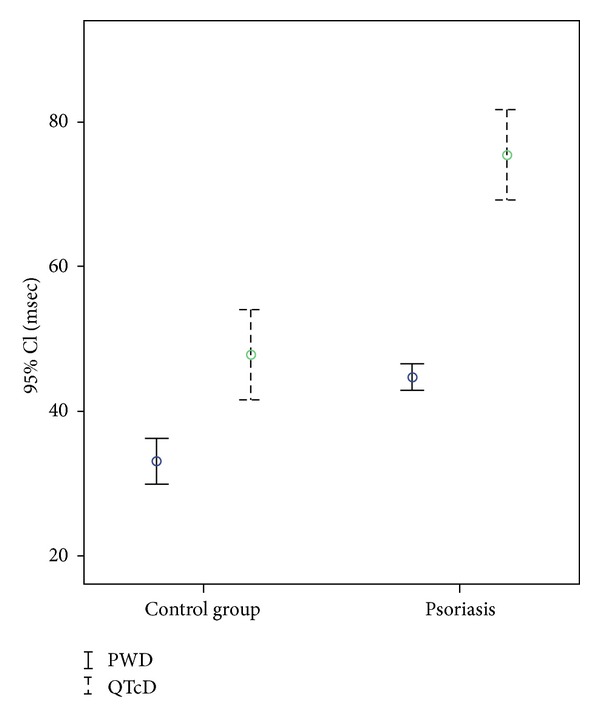
Error box of P wave dispersion and QTc dispersion in patients with psoriasis and control group.

Mean disease duration was 129,4 ± 83,9 (range, 3–360) months, and PASI ranged from 0 to 34,0 (mean ± SD; 7,6 ± 6,7). Seventy-seven patients (81,9%) had plaque type, 8 (8,5%) patients had palmoplantar type, and 9 (9,6%) patients had pustular type psoriasis. There were no significant differences between psoriasis patients and control subjects in respect to age, gender, body mass index, blood pressures, smoking, and lipid profiles (Table 1). Of the diastolic function parameters, DT and IVRT were significantly longer among psoriasis patients. However, frequency of diastolic dysfunction was not significantly increased (Table 1). Compared to control group, psoriatic patients had significantly shorter Pmax and Pmin durations, longer QTcmax, and greater PWD and QTcD (Table 2, Figure 1). QTcD and PWD were significantly correlated with disease duration (*r* = 0,693, *P* < 0,001, and *r* = 0,368, *P* = 0,003, resp.) but not with any other clinical or echocardiographic parameter. In addition DT and IVRT were significantly correlated with age (*r* = 0,469, *P* = 0,001, and *r* = 0,568, *P* < 0,001, resp.). There were no significant correlations between PASI and PWD and QTcD.

## 5. Discussion 

In this study, we found that both PWD and QTcD are increased in psoriasis patients compared to healthy subjects. In addition, they had longer DT and IVRT.

Several studies have demonstrated that cardiovascular diseases and their associated risk factors like hypertension, diabetes, hyperlipidemia, obesity, and smoking are more common in patients with psoriasis than in the general population [19, 20]. Intrinsic associated risks and/or psychological impact of psoriasis driving risky behaviors such as obesity and smoking may increase cardiovascular risk [21, 22]. 

However, there is scarcity of data on rhythm abnormalities and conduction disturbances in psoriatic patients, despite a quite clear connection between arrhythmia occurrence and chronic inflammatory processes [23, 24].

Systemic inflammatory response and oxidative stress are the most important mechanisms in the development of psoriasis [2, 3]. Furthermore, recent studies have also demonstrated an inflammatory background of ventricular arrhythmias and the possible implication of inflammation and oxidative stress in pathogenesis of AF [13–15, 18]. 

P-wave duration and P wave dispersion are the most important ECG markers used to evaluate the risk of atrial arrhythmias [25]. Several studies showed that PWD has a predictive value for AF in patients with various conditions [26, 27]. Various studies have demonstrated that increased QT interval and QT dispersion may increase the incidence of sudden death and ventricular tachycardia in many cardiovascular conditions and noncardiac diseases. It has been shown that PWD and QT interval and QT dispersion were increased in autoimmune diseases such as rheumatoid arthritis, SLE, and systemic sclerosis [28–31]. 

Inflammation associated with psoriasis may have an effect on increased PWD and QTcD. However, lack of measurement of inflammatory markers in our study limits this conclusion. Despite many shortcomings, PASI is still the most commonly used score for the assessment of severity of psoriasis [32]. Severity of the disease may reflect intensity of inflammation and therefore may be associated with PASI. Lack of significant correlation between PWD and QTcD with PASI may be due to relatively low PASI levels in our study population. 

P wave dispersion and QTD have been shown to be increased in LV diastolic dysfunction [33, 34]. It has been reported that psoriasis may accompany mild diastolic dysfunction [35]. Although we could not find a significant association between frequency of diastolic function and PWD and QTcD, still there may be a possible contribution of diastolic dysfunction parameters to the increased PWD in psoriasis.

Increased sympathetic activity may be another possible mechanism for increased PWD and QTcD in psoriasis. Sympathetic activity has been linked to PWD and QT-dispersion [36, 37]. Compared with control subjects, psoriasis patients displayed a blunted increase in heart rate and a sharper increase in diastolic blood pressure during stress examination [38].

## 6. Limitations

Small number of study patients and lack of long-term clinical followup are the major limitations of our study. Another important limitation is lack of measurements of markers for inflammation. Automated ECG measurements were not available and manual calculation of P wave and QT measurements may be criticized. Whether increased PWD and QTcD in patients with psoriasis predicts poorer clinical outcomes or mandates any special treatments warrants further study. 

## 7. Conclusions

P wave dispersion and QTcD are increased in psoriasis patients and correlated with disease duration. In addition PWD correlated with diastolic function parameters of IVRT and DT. Our findings suggest that increased PWD and QTd are potentially useful, simple, and noninvasive methods for the early detection of subclinical cardiac involvement in patients with especially long-lasting psoriasis. We suggest that long-lasting psoriasis patients may be considered for referral for cardiovascular evaluation. Larger and longer-term studies are necessary to evaluate clinical implications of our findings.

## Figures and Tables

**Table 1:** Comparison of clinical, laboratory, and echocardiographic variables between psoriasis patients and healthy subjects.

	Psoriasis patients (*n* = 94)	Control group (*n* = 51)	*P* value
Age (years)	35,8 ± 13	36,9 ± 10,4	0,751
Male (*n*, %)	44 (47%)	33 (65%)	0,206
Smoking (*n*, %)	24 (25%)	18 (35%)	0,443
BMI (kg/m^2^)	25,6 ± 5,9	23,8 ± 3	0,245
Systolic BP (mmHg)	121,2 ± 12,9	119,7 ± 11,2	0,66
Diastolic BP (mmHg)	78,8 ± 9,2	77 ± 6,8	0,471
Total cholesterol (mg/dL)	167,6 ± 47,4	174 ± 36,4	0,582
LDL-C (mg/dL)	97 ± 35,5	103,9 ± 31,5	0,484
HDL-C (mg/dL)	45,8 ± 13,3	44,4 ± 9,8	0,689
Triglyceride (mg/dL)	152,6 ± 83,7	153 ± 88,6	0,985
LVDD (mm)	45,9 ± 3,3	44,2 ± 5,1	0,121
LVSD (mm)	31,1 ± 3,9	32,1 ± 3,1	0,462
LVEF (%)	61,1 ± 3,8	62,6 ± 3,9	0,155
IVS (mm)	9,9 ± 1,4	9,5 ± 1	0,216
PW (mm)	9 ± 1,3	8,9 ± 0,9	0,7
LA diameter (mm)	33,8 ± 4,4	33,9 ± 3,8	0,935
E/A	1,29 ± 0,3	1,39 ± 0,3	0,271
DT (msec)	195,9 ± 29,7	176,4 ± 11	**0,011**
IVRT (msec)	91,6 ± 14,7	80,1 ± 10	0,04
Diastolic dysfunction (*n*, %)	8 (8,5%)	0	0,309

BP: blood pressure, BMI: body mass index, LVDD: left ventricular diastolic diameter, LVSD: left ventricular systolic diameter, LVEF: left ventricular ejection fraction, IVS: interventricular septum, PW: posterior wall, LA: left atrium, DT: deceleration time, and IVRT: isovolumetric relaxation time.

**Table 2:** Comparison of P wave and QTc parameters and heart rate between psoriasis patients and healthy subjects.

	Psoriasis patients (*n* = 94)	Control group (*n* = 51)	*P* value
Pmax (msec)	77,8 ± 9,1	82,9 ± 10,9	0,064
Pmin (msec)	35,9 ± 6,1	52,6 ± 10,8	**<0,001**
PWD (msec)	41,9 ± 7,6	30,3 ± 7,2	**<0,001**
Heart rate (bpm)	77,8 ± 13,7	77,1 ± 13,4	0,99
QTcmax (msec)	488,8 ± 87,2	421,2 ± 52,9	**0,01**
QTcmin (msec)	397,2 ± 59,1	373,4 ± 47,6	0,19
QTcD (msec)	75 ± 18,3	47,8 ± 10,2	**<0,001**

PWD: P wave dispersion and QTcD: QT corrected dispersion.
